# Dual Dependence of Cryobiogical Properties of Sf21 Cell Membrane on the Temperature and the Concentration of the Cryoprotectant

**DOI:** 10.1371/journal.pone.0072836

**Published:** 2013-09-04

**Authors:** Jianye Wang, Kaixuan Zhu, Gang Zhao, Jian Ren, Cui Yue, Dayong Gao

**Affiliations:** Institute of Biomedical Engineering, Department of Electronic Science and Technology, University of Science and Technology of China, Hefei, Anhui, People’s Republic China; The Ohio State University, United States of America

## Abstract

The Sf21 cell line is extensively used for virus research and producing heterologous recombinant proteins. To develop optimal strategies for minimizing cell injury due to intracellular ice formation and excessive volume shrinkage during cryopreservation, the fundamental transport properties including the osmotic inactive volume (*V_b_*), the hydraulic conductivity (*L_p_*), and the glycerol permeability (*P_s_*) of Sf21 cell membrane at 25, 15, 5 and −2°C were characterized using a micro-perfusion chamber. The effects of temperature on the hydraulic conductivity and the glycerol permeability of Sf21 cell membrane, reflected by the activation energies, were quantitatively investigated. It was found that the hydraulic conductivity decreases along with the increase of the final CPA concentration at a given temperature, and quantitative analysis indicates that the hydraulic conductivity has a significant linear attenuation along with the increase of the concentration of glycerol. Therefore, we incorporate the concentration dependence of the hydraulic conductivity into the classic Arrhenius relationship by replacing the constant reference value of the hydraulic conductivity at the reference temperature with a function that is linearly dependent on the CPA concentration. Consequently, the prediction of the Arrhenius relationship is improved, and the novel Arrhenius relationship could be very important to the development of optimal strategies for cell cryopreservation.

## Introduction

The Sf21 cell line, derived from the ovary of the fall armyworm (*Spodoptera Frugiperda*) by Vaughnet at 1977 [1], is a host of many viruses, and when combined with baculoviruses, is a powerful platform technology that is widely used for the manufacture of viral particles and heterologous recombinant proteins [2], [3], [4]. This robust expression system is able to produce large amounts of different proteins, with applications from basic research (protein-protein interaction, multiple-affinity protein purification, ultrasensitive mass spectrometry, and protein structures) [5] to clinical medicine (routine diagnostic tests, therapeutic protein drugs for various diseases, etc.) [6], [7], [8].

The maintenance of viable insect cell line cultures is a time-consuming and very expansive program. Long-term preservation of the insect cell lines through cryopreservation is considered to be a better alternative to meet the ever-increasing demands. Cryopreservation is an enabling technology for cell and tissue banking because metabolic activities can be arrested and life temporarily suspended at cryogenic temperatures. However, cells could be damaged upon freezing by extracellular ice formation, intracellular ice formation (IIF) [9], [10] and excessive volume shrinkage [11] due to the elevation of solute concentration in the surrounding of cells. Cryoprotective agents (CPAs) are commonly used to minimize the damage to cells during freezing. Transport properties of cell membrane to water and cryoprotective agents are known to play a fundamental role in cryopreservation. To optimize the freezing and thawing processes as well as the addition and removal of CPAs, the transport properties of cell membranes should be determined. Transport properties of cell membrane can be measured by a number of methods, including the photomicroscopic technique [12], the microscopic stopped flow technique [13], the microdiffusion chamber technique [14], the micropipette perfusion [15], the microfluidic perfusion system [16], and the perfusion microscope system [17], [18]. The photomicroscopic technique determines the distribution of transport properties of the cell membrane, and is an impractical and irreversible method due to the deviation of the individual cell [12]. The microscopic stopped flow method controls the concentration change of the extracellular solution via the mixing of two fluid streams, and thus is able to record the transient cell volume change [13]. The microdiffusion chamber technique minimizes cell damage during the measurement, but it may generate a heterogeneous exchange rate of mass in channel due to its cell blocking geometry [14]. Inspired by different designs of the microdiffusion chamber [14], the micropipette perfusion system [15], the microfluidic perfusion system [16], and the perfusion microscope system [17], [18], we developed a low-cost, easy-to-use microperfusion chamber for the investigation of cell osmotic responses.

The osmotic properties of the Sf21 cell membrane were experimentally studied using this micro-perfusion chamber. The membrane permeability coefficient to water (*L_p_*), the membrane permeability coefficient to glycerol of different concentration (*P_s_*), and the activation energies of water and glycerol permeability coefficients at 25, 15, 5 and −2°C have been investigated. Furthermore, the effects of the temperature and the CPA concentration on the transport properties of the Sf21 cell membrane were analyzed and a novel Arrhenius relationship was proposed for predicting the osmotic behaviors of cell membrane during cryopreservation.

## Materials and Methods

### Theory of Cell Membrane Transport Model

Kedem and Katchalsky proposed the *K*-*K* model to descript the transport of both water and the CPA through the cell membrane [19], and the *K*–*K* formalism can be described as follows:
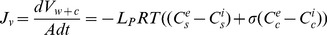
(1)

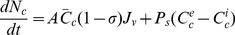
(2)where *J_v_* is the total volume flux, *V_w+c_* is the volume of water and solute volume, *N_c_* is the number of moles of the permeating solution, *A* is the area of the cell, and *C* is osmolarity of the solution. The superscript *i* and *e* represent the intra- and extracellular, respectively; the subscript *s* and *c* represent the salt and the CPA, respectively. 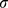 is the reflection coefficient. 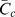 is the mean of the osmolarity of the CPA.

The two-parameter (2-*p*) model is often used to describe the cell volume response during osmotic shift in a ternary system [20]. In which, water and CPA flux across the plasma membrane can be described as follows [14], [15], [21]:
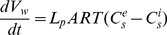
(3)

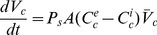
(4)


The *2-p* model employs only two parameters *L_p_* and *P_s_* to describe the flux of water and CPA through the cell membranes. 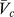 is the partial molar volume of CPA.

The water permeability at different temperatures obeys the following Arrhenius relationship [12], [22], [23]:
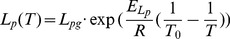
(5)where *E_Lp_* is the activation energies of water permeability coefficients and *L_pg_* is water membrane permeability coefficient at a reference temperature (usually, at *T_0_* = 273.15 K), *R* is the universal gas constant. Taking the natural logarithm in the equation simultaneously, it turns to be
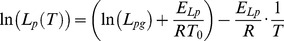
(6)


### Cell Culture and Sample Preparation

Sf21 cell line was obtained from Prof. Gang Cai as a gift [24], and SF21 cells were cultured using SF900II SFM (Gibco) without fetal bovine serum at 28°C, and the cells were harvested when the number of cells was about 1×10^7^/ml. The cells were re-suspended in PBS and centrifuged at 600 g for 5 min.

### Experimental Procedure

**Figure 1:**
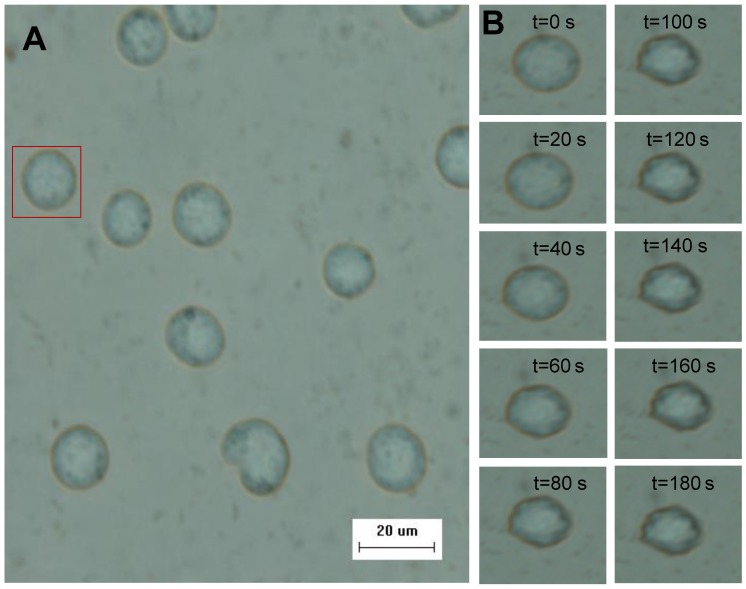
The micro-perfusion system. A Schematic diagram showing the arrangement of the major components. B Photograph of the micro-perfusion chamber mounted on the microscope stage.

The micro-perfusion system developed based on those of Takamatsu *et al*. [17], [18], Gao *et al.* [15], [16], and McGrath *et al.* [14], as shown in Figure 1, was applied to study cell osmotic response in this study. The micro-perfusion chamber was mounted on the inverted microscope (Ti-FL, Nikon, Japan). SF21 cells were immobilized after the suspending cells were injected to the micro-channel. The cell volume response during the experiments was recorded to the computer *via* a CCD camera (DS-Ri1, Nikon, Japan) and a digital camera (DS-U3, Nikon, Japan). The image frames were extracted from the videos and saved to prepare for image processing.

In this experiment, we observed the cell volume responses after being transferred from PBS to 3×PBS, and from PBS to the solutions of different glycerol concentration at 25, 15, 5 and −2°C, respectively.

### Data Collection and Analysis

The video was converted into the image frames and a series of images with a 10s-interval were extracted from the images frames. The Sf21 cells were assumed to be spherical, and we could calculate the volumes of Sf21 cells thought the area of the cross section. The non-spherical shaped cells and the cells with over-small or over-large volumes were discarded. Then the parameter was incorporated into equations (1)-(6) to fit the membrane permeability coefficients. In order to obtained a high precision fitting process, a universal self-adaptive time-varying function for the extracellular concentration profile was used [25]. Parameters of the cells and the fitted permeability coefficients were presented in the form of the Mean±Standard Deviation.

## Results and Discussion

**Figure 2:**
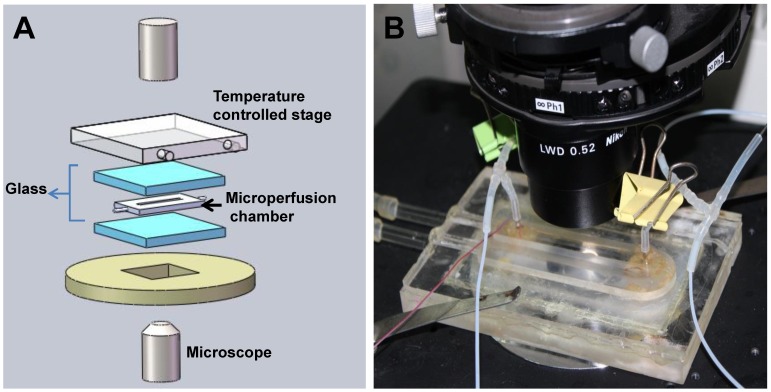
The morphology and volume changes of representative cell. A The Sf21 cells were immobilized on the micro-channel. B The volume changes of a typical cell during the osmotic shift from PBS to 3×PBS solution at −2°C.

Figure 2A shows the Sf21 cells adhered to the micro-channel in the initial state at −2°C, and the transient response of a representative cell (indicated by a red box in the left figure) during the osmotic shift from PBS (291 mOsm) to 3×PBS (784 mOsm) at −2°C was shown in Figure 2B at a 10 second intervals. The measured cell volume changes at four different temperatures and the corresponding fitting processes are comparatively shown in Figure S1. Table 1 summarizes the values of the inactive cell volume (*V_b_*) and the hydraulic conductivity at four different temperatures. The mean value of the inactive cell volume is 0.518 and the standard error is 0.089, n = 25. The *V_b_* of the Sf21 is similar to mammalian ovarian tissues (0.5 *V_0_*) [26], [27], [28].

**Table 1:** Cryobiological parameters for Sf21.

Temperature(°C)	*V_b_/V_0_*(%)	*L_p_*	
		(10^−14^m/Pa/s)	(µm/atm/min)
25(n = 12)	54.6±9.1	1.112±0.334	0.068±0.020
15(n = 8)	50.6±11.8	0.910±0.277	0.055±0.017
5(n = 11)	51.3±4.1	0.449±0.090	0.027±0.006
−2(n = 7)	50.8±6.1	0.368±0.101	0.023±0.006

**Figure 3:**
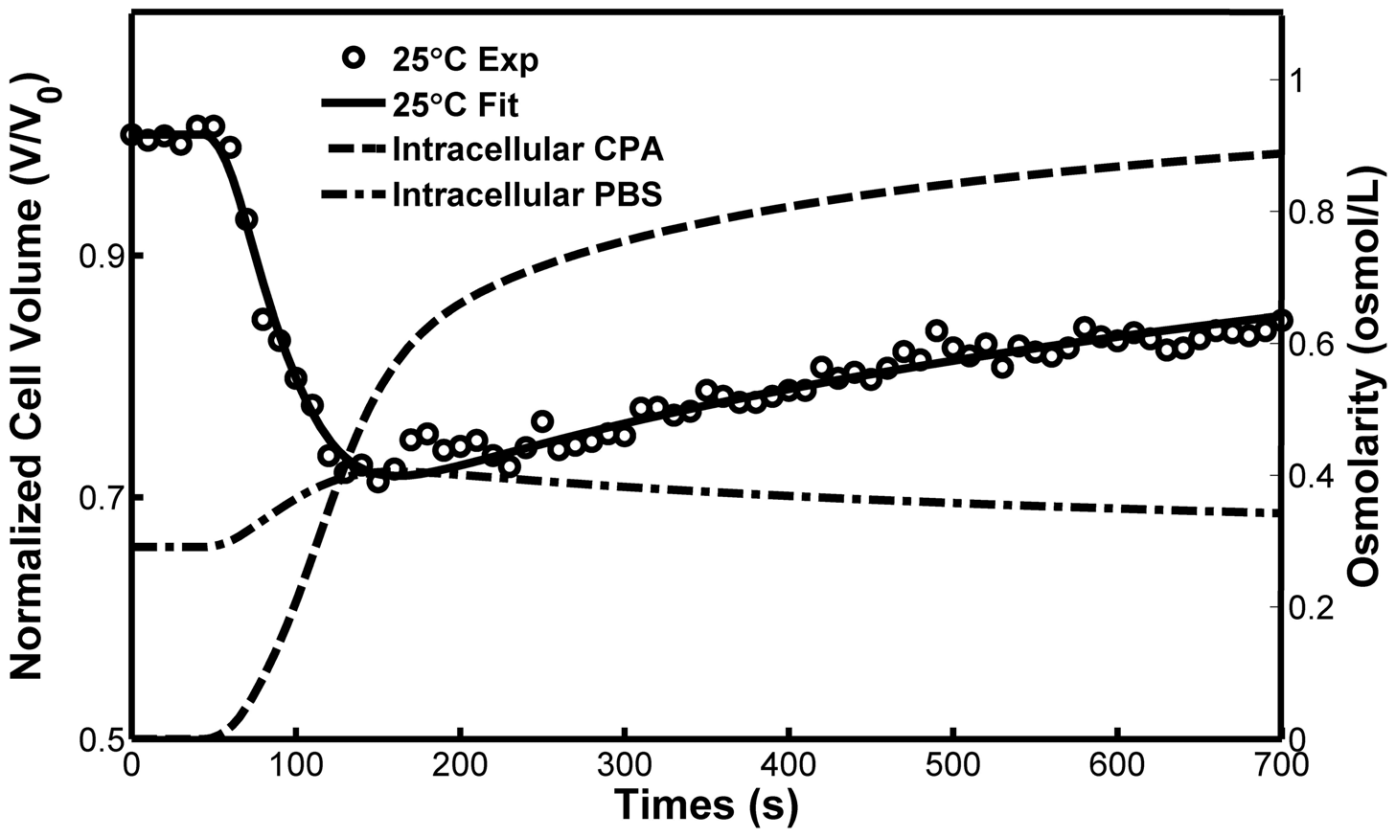
Volume change of a Sf21 cell and osmolarity shift of CPA and PBS in the intracellular solution during the solution from the PBS to PBS solution with 1.0 M glycerol at 25°C.

The measured cell osmotic responses and the curve-fitting processes using the 2-*p* model after the addition of 1.0 M glycerol at four different temperatures are comparatively shown in Figure S2. Figure 3 presents volume change of a Sf21 cell and osmolarity shift of CPA and PBS in the intracellular solution during the solution from the PBS to PBS solution with 1.0 M glycerol at 25°C. The cell membrane permeability coefficients fitted using both the *K*-*K* and the 2-*p* models for the addition of 1.0, 1.5 and 2.0 M glycerol are listed in tables 2, 3, 4, respectively. Apparently, individual variation of the results of the *K-K* and the *2-p* models are quite a few marked-pronounced. In the *K-K* model, the hydraulic conductivity, the solute permeability coefficient, and the reflection coefficient, are used to characterize the cell membrane. In order to describe the water and solute flux interaction across the cell membrane, the reflection coefficient was introduced [19]. It has been pointed out that the *K*-*K* model does not account for any membrane moderated transport and possible contributions of water channels. To fully consider the contribution of the water channel, the 2-*p* model was suggested, in which the interaction between water and CPA was neglected and their transport through the cell membrane is regarded to be independent [20]. In this study, all the permeability coefficients fitted using both the *K*-*K* and the 2-*p* models are presented, and the analysis are based on the parameter of *2-p* model.

**Table 2:** The cell membrane permeability coefficients of Sf21 for 1.0 M glycerol.

Temperature (°C)	K-K model parameters			2-p model parameters	
	*L_p_*(10^−14^m/Pa/s)	*P_s_*(10^−8^ m/s)	σ	*L_p_*(10^−14^m/Pa/s)	*P_s_*(10^−8^ m/s)
25(n = 9)	0.620±0.176	0.619±0.314	0.98±0.04	0.748±0.524	0.648±0.284
15(n = 11)	0.533±0.356	0.501±0.192	0.91±0.16	0.658±0.551	0.395±0.160
5(n = 9)	0.368±0.175	0.101±0.050	0.94±0.14	0.335±0.97	0.107±0.051
−2(n = 7)	0.334±0.124	0.047±0.017	0.93±0.16	0.274±0.047	0.038±0.010

**Table 3:** The cell membrane permeability coefficients of Sf21 for 1.5 M glycerol.

Temperature (°C)	K-K model parameters			2-p model parameters	
	*L_p_*(10^−14^m/Pa/s)	*P_s_*(10^−8^ m/s)	σ	*L_p_*(10^−14^m/Pa/s)	*P_s_*(10^−8^ m/s)
25(n = 11)	0.422±0.275	0.759±0328	0.92±0.15	0.654±0.430	0.682±0.298
15(n = 9)	0.429±0.126	0.349±0.144	0.99±0.02	0.429±0.131	0.397±0.124
5(n = 8)	0.203±0.051	0.081±0.041	0.99±0.01	0.195±0.051	0.117±0.048
−2(n = 11)	0.025±0.089	0.045±0.034	0.86±0.23	0.173±0.016	0.046±0.032

**Table 4:** The cell membrane permeability coefficients of Sf21 for 2.0 M glycerol.

Temperature (°C)	K-K model parameters			2-p model parameters	
	*L_p_*(10^−14^m/Pa/s)	*P_s_*(10^−8^ m/s)	σ	*L_p_*(10^−14^m/Pa/s)	*P_s_*(10^−8^ m/s)
25(n = 9)	0.258±0.080	0.819±0.195	0.99±0.01	0.269±0.077	0.760±0.123
15(n = 7)	0.231±0.113	0.672±0.135	0.98±0.03	0.225±0.074	0.538±0.082
5(n = 12)	0.089±0.028	0.132±0.062	0.96±0.03	0.109±0.038	0.158±0.068
−2(n = 10)	0.097±0.036	0.096±0.016	0.98±0.05	0.098±0.037	0.098±0.016

In previous studies, the effect of the CPA concentration on the hydraulic conductivity has not been fully established [29]. Hempling *et al.* [30] found that the hydraulic conductivity was sharply reduced by 50% with the concentration of dimethyl sulfoxide (DMSO) increasing from 0.6 M to 1.2 M in megakaryocytopoietic cells, and there is no further reduction in the hydraulic conductivity with the increase of the DMSO concentration. However, McGrath *et al.* [31] got the conflict conclusion for the mouse oocytes research, where the *L_p_* value of the mouse oocytes increased by 70% in the presence of 1.5 M DMSO or 1,2-propanediol. In this study, the Sf21 cell was used as a model cell, and the effects of the temperature and the CPA concentration on its hydraulic conductivity were quantitative investigated.

**Figure 4:**
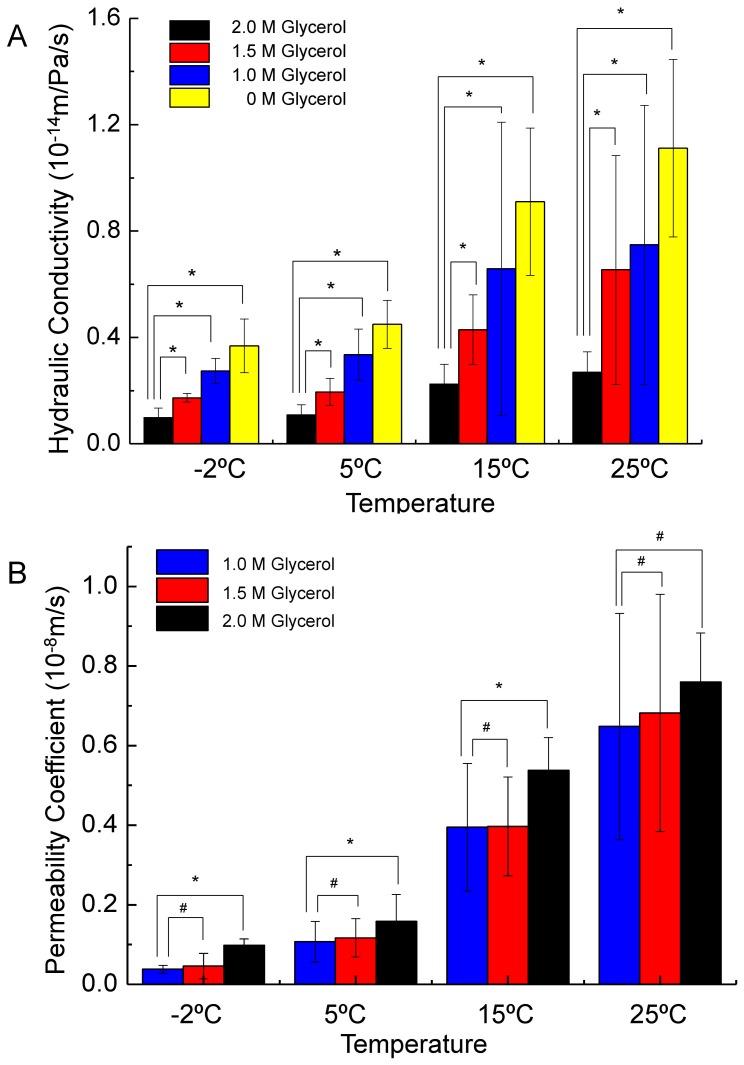
The hydraulic conductivity and permeability coefficient of Sf21 cell membrane. A The hydraulic conductivity of Sf21 cell membrane in the presence of 0, 1.0, 1.5, 2.0 M glycerol at 25, 15, 5 and −2°C, respectively. B The CPA permeability coefficient of Sf21 cell membrane in the presence of 1.0 M, 1.5 M, 2.0 M glycerol at 25, 15, 5 and −2°C, respectively. #, there is no significant difference in student *t*-test (*p>*0.05); *, there is significant difference in student *t*-test *(p*<0.05).

**Figure 5:**
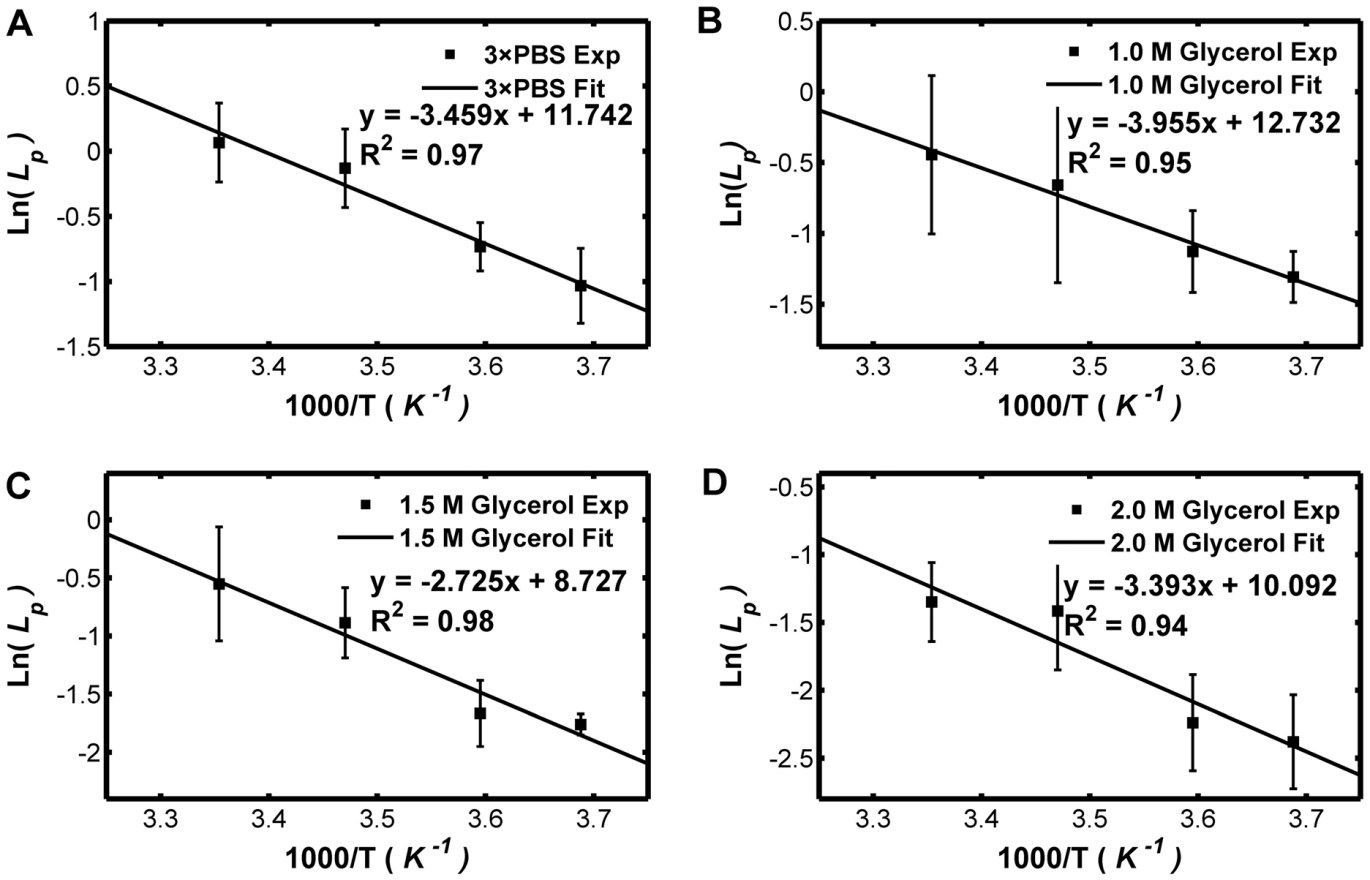
Arrhenius plot of the hydraulic conductivity for solution in the presence of 0, 1.0, 1.5, 2.0 M glycerol. The natural logarithms of the hydraulic conductivity (10^−14^m/Pa/s) are linearly dependent to the 1000 fold of the reciprocal of the absolute temperature.

**Figure 6:**
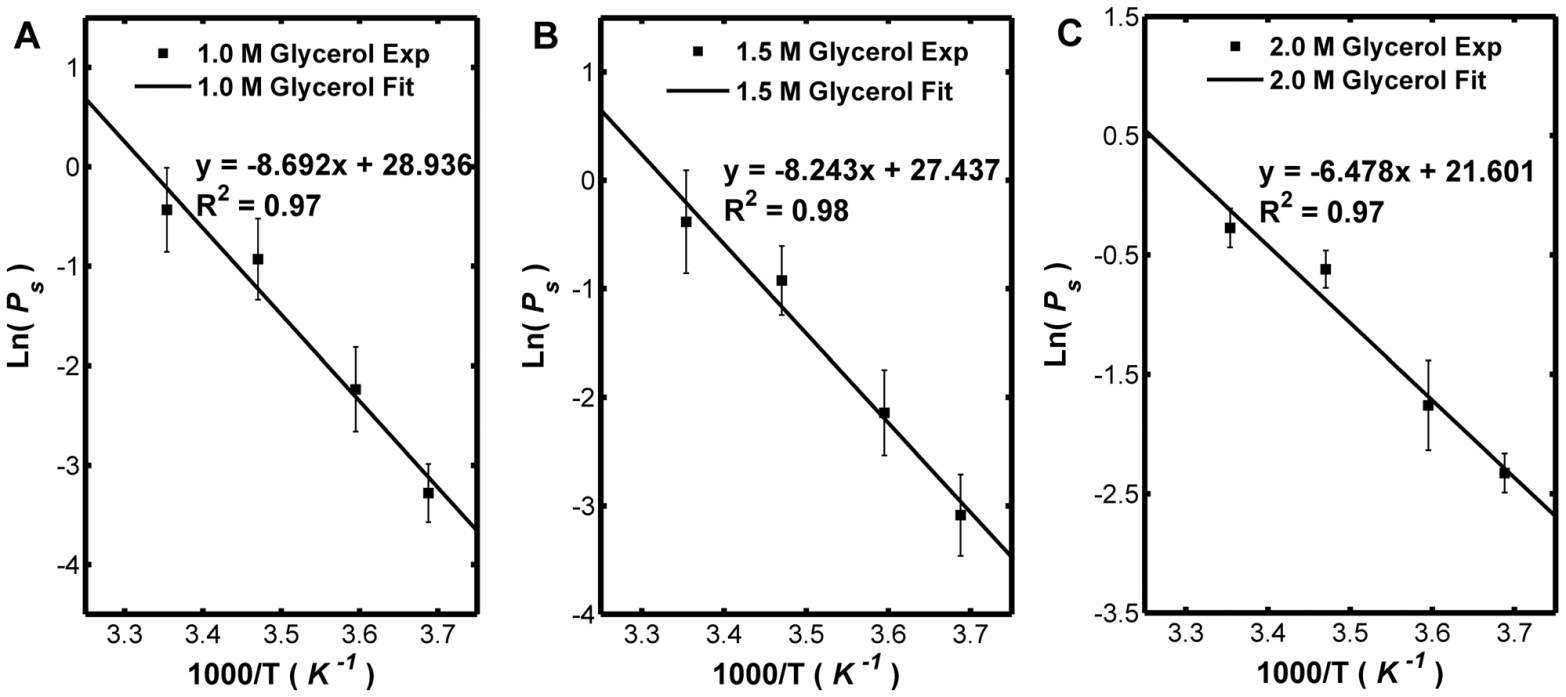
Arrhenius plot of the hydraulic conductivity for solution in the presence of 1.0, 1.5, 2.0 M glycerol. The natural logarithms of the glycerol permeability coefficient (10^−8 ^m/s) are linearly dependent to the 1000 fold of the reciprocal of the absolute temperature.

**Figure 7:**
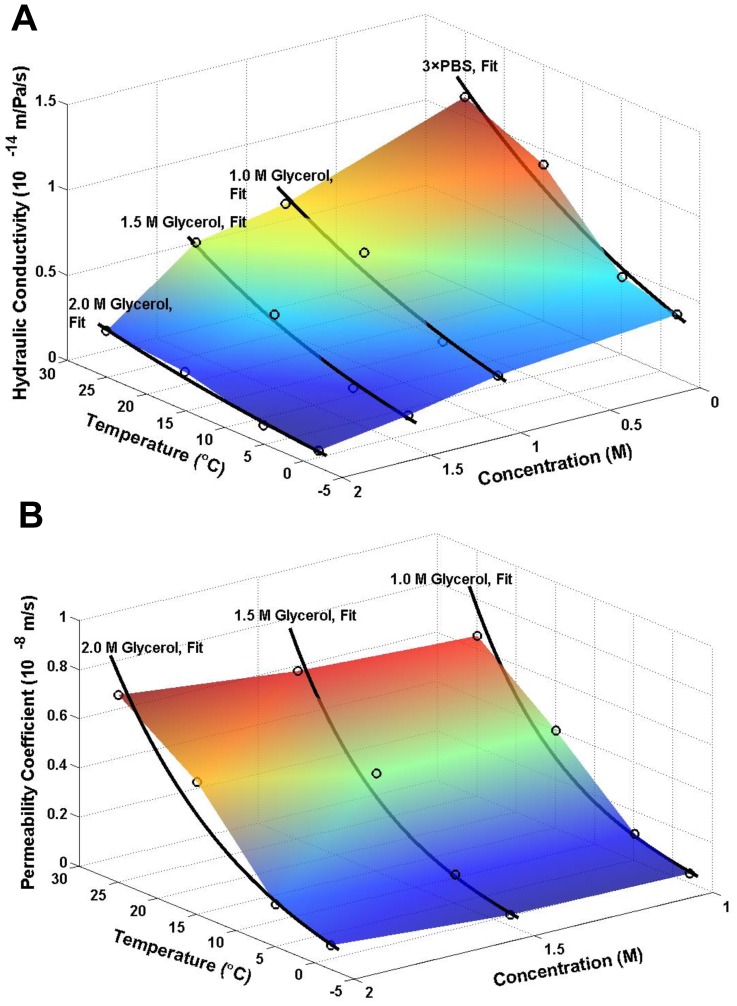
The overview of the dependences of the hydraulic conductivity and permeability coefficient of Sf21 cell on temperature and the glycerol concentration. A Dependences of the hydraulic conductivity of Sf21 cell on temperature and the glycerol concentration. The circles, experimental data; the curves, predictions using the Arrhenius formula. B Dependences of the CPA permeability coefficient of Sf21 cell on temperature and the glycerol concentration. The circles, experimental data; the curves, predictions using the Arrhenius formula.

Figure 4 shows the transport properties of Sf21 cell membrane for different concentration of glycerol, from which it can be seen that the *L_p_* value sharply decrease along with the increase of the CPA concentration at high temperature, while such decrease is comparatively slight at low temperatures. However, the *P_s_* values do not apparently changes with the CPA concentration for all the temperatures studied. Figure 5 and 6 show that the dependence of the hydraulic conductivity and the glycerol permeability coefficient on temperature was analyzed by least-square curve-fitting and the dependence was validated by the classic Arrhenius relationship. The *E_Lp_*, *L_pg_*, *E_Ps_ and P_sg_* values of the Sf21 were determined according to the linear regression, and they were listed in Table 5. Apparently, the *L_pg_* value of Sf21 decreases along with the increase of the glycerol concentration, while the *E_Lp_* doesn’t have the responding decline. Besides, the *P_sg_* value increases with the increase of the glycerol concentration. The dependences of the hydraulic conductivity and the glycerol permeability coefficient of Sf21 cell membrane on temperature and the final glycerol concentration were shown in Figure 7.

**Table 5:** The *E_Lp_*, *L_pg_*, *E_Ps_ and P_sg_* of the Sf21 for different osmotic shifts.

Osmotic Shift	*E_Lp_* (kJ/mol)	*L_pg_* (10^−14^m/Pa/s)	*E_Ps_* (kJ/mol)	*P_sg_* (10^−8^m/s)
PBS → 3×PBS	29.220	0.395	–	–
PBS → PBS+1.0 M glycerol	26.727	0.297	70.667	0.056
PBS → PBS+1.5 M glycerol	34.911	0.175	67.013	0.065
PBS → PBS+2.0 M glycerol	27.396	0.101	52.664	0.121

**Figure 8:**
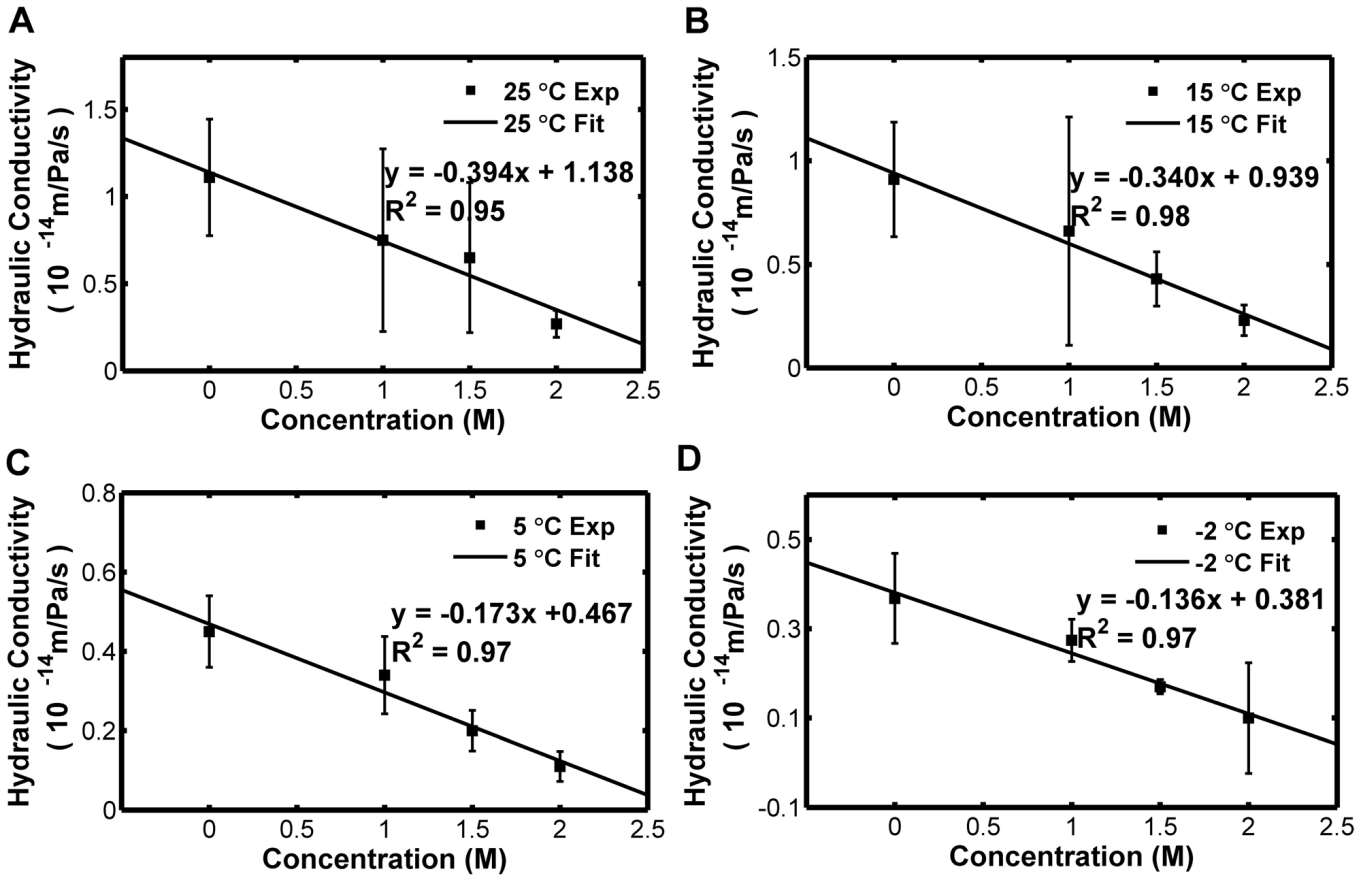
Dependence of the hydraulic conductivity of Sf21 cell membrane on the final glycerol concentration at 25, 15, 5 and −2°C, respectively.

**Figure 9:**
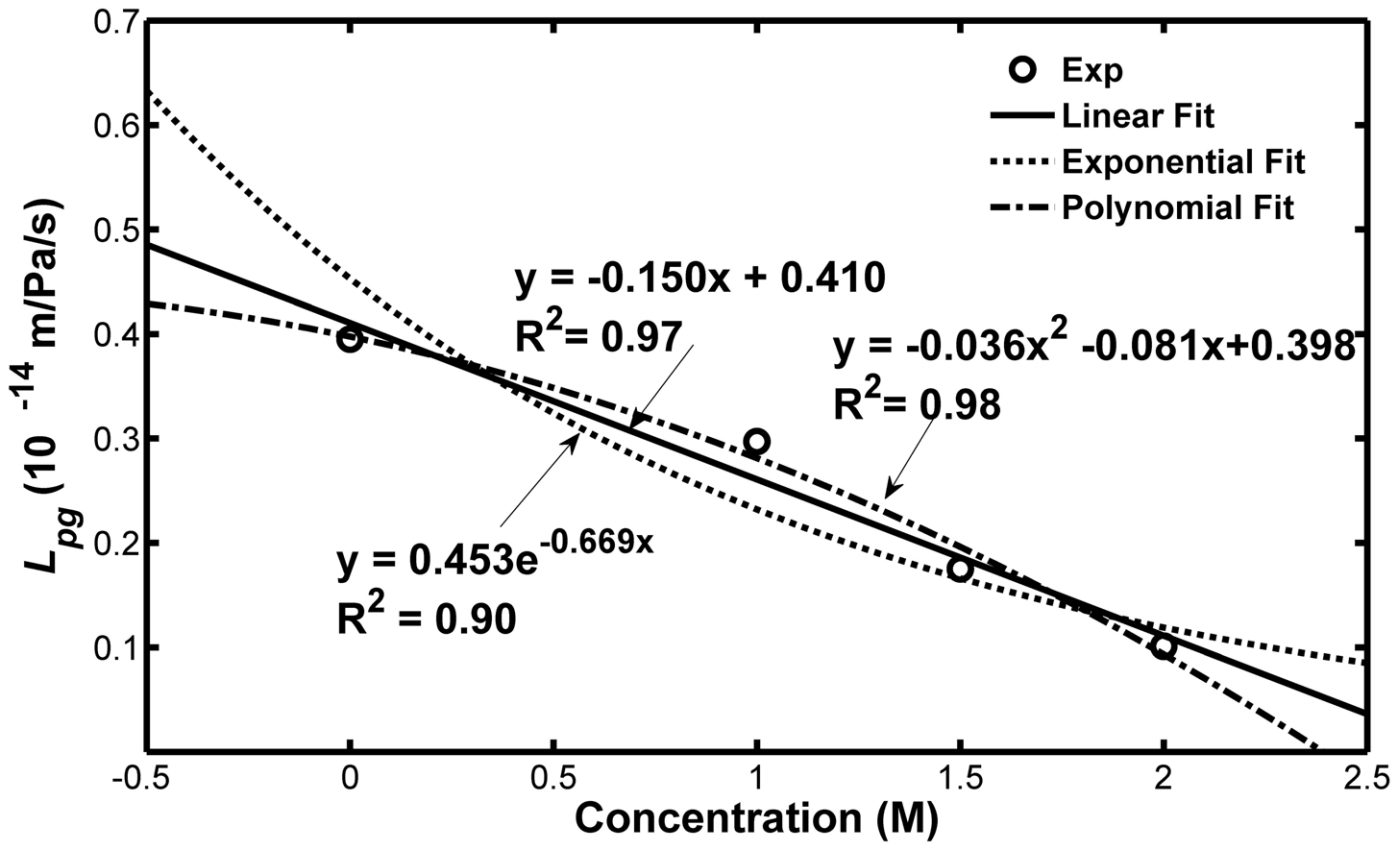
Dependence of the reference value of the hydraulic conductivity (*L_pg_*) of Sf21 cell on the glycerol concentration. The results indicate that the linear fitting is better than the exponential fitting.

McGrath *et al.* [32] found that the hydraulic conductivity seemingly reduced exponentially in the presence of increased concentration of CPA based on their data. However, it was found that the hydraulic conductivity has a significantly linear decrease with the increase of the glycerol concentration, as shown in Figure 8. From Figure 9, the reference value of the hydraulic conductivity of Sf21 also has a significantly linear decrease with the increase of the final glycerol concentration. Although the polynomial fitting seems to be even better than the linear, it was not recommended by this study due to the difficulty in demonstrating the mechanism.

**Figure 10:**
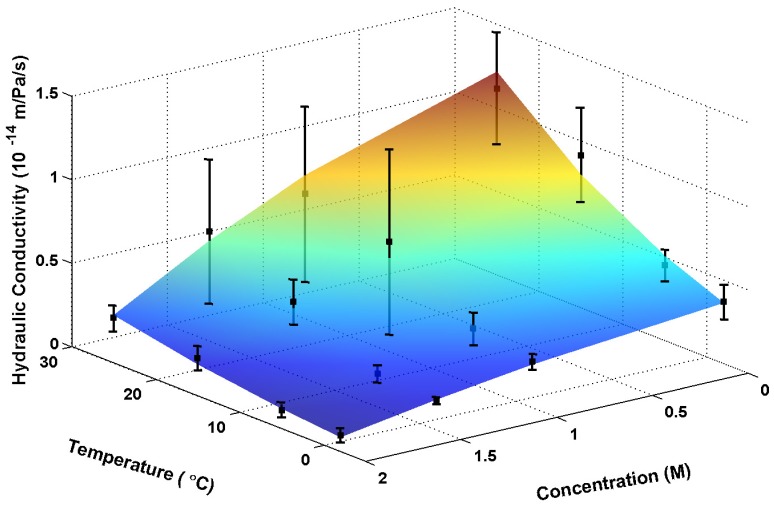
Dependences of the hydraulic conductivity on glycerol concentration and temperature predicted using modified Arrhenius relationship. The black boxes represent the experimental data (25, 15, 5 and −2°C; 0, 1, 1.5 and 2 M glycerol), and the vertical lines indicate the standard error.

It was assumed that the dependence of the *L_pg_* on the molar concentration of the CPA could be described using the following equation,
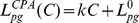
(7)where the superscript *CPA* means there is CPA in the solution, and the superscript *0* means there is no CPA in the solution. *C* is the concentration of the CPA. Then eq. (7) is incorporated into eq. (5) to reflect the dependences of the hydraulic conductivity on both the temperature and the CPA molar concentration. Finally, the *L_p_* values of Sf21 at any temperature and the given CPA concentration could be predicted by the following equation,



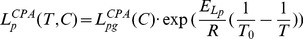
(8)The predictions of Eq. (8) and the experimental data were comparatively shown in figure 10, as can be seen that they agree well with each other. This implies that eq. (8) is applicable for Sf21 cells.

In eq. (8), the *L_p_* values of Sf21 are well described with the molar concentration of CPA and the temperature. In aqueous glycerol solution, the solution viscosity steadily climbs up with increasing concentration according to the WLF model [33], [34]. Beside, the high molar glycerol concentration indicates the water have a small proportion in the solution. Dual effects may indicate that the hydraulic conductivity has a significantly linear decrease with the increase of the final glycerol concentration. While, there are still no definitive conclusions on the relationship between the CPA and the water channels.

Figure S3 shows the glycerol permeability coefficient of Sf21 cell with the concentration of the glycerol at 25, 15, 5 and −2°C, respectively. It seems that the glycerol permeability coefficient increases with the increase of the CPA molar concentration, while no further quantitative relationship is found due to the limited experimental data.

There is a dynamic equilibrium in cell volume due to the water efflux as glycerol transported into the cell and water driven by the chemical potential [35], and then the volume change could been predicted using the cryobiological parameters in the experiment. We implement the new Arrhenius relationship in predicting the transient cell volume suffered the osmotic shifts. Figure S4 shows the predicted cell volume changes of Sf21 after various osmotic shifts at 25°C using the permeability coefficients measure by this study (from PBS to 1.0, 1.5 and 2.0 M glycerol solutions). While, the researcher hypothesized the transport properties are independent to the glycerol concentration. This novel Arrhenius relationship induces the relationship of cryobiological properties, and it could be very important to the development of optimal strategies for cell cryopreservation.

## Conclusion

The osmotically inactive volume, the hydraulic conductivity and the glycerol permeability coefficient of Sf21 cells were determined experimentally at 25, 15, 5 and −2°C. The parameters of both the *K-K* model and the 2-*p* model of Sf21 cells were fitted in the presence of 1.0, 1.5, 2.0 M glycerol at 25, 15, 5 and −2°C. We found that *L_p_* sharply decreases with the increase of the CPA concentration at high temperature, while the reduction is relatively less at lower temperatures. The *L_pg_* of Sf21 cells decreases with the increase of glycerol concentration in the solution, while such trend was not observed for the *E_Lp_* of Sf21. The *E_Ps_* and *P_sg_* increase slightly with the increase of the glycerol concentration. We proposed that the hydraulic conductivity has a significant linear correlation with the concentration of glycerol for the Sf21 cells and the *L_g_* values correlate well with temperature and the molar concentration of glycerol. The volume changes were predicted using the cryobiological parameters in the experiment. The concentration dependence of the hydraulic conductivity was incorporated into the classic Arrhenius relationship by replacing the constant reference value of the hydraulic conductivity at the reference temperature with a function that is linearly dependent of the CPA concentration. Further comparison between the predictions of the extended formula with the experimental data indicates that this formula was adequate at least for the Sf21 cells.

## Supporting Information

Figure S1 **Volume responses of Sf21 cell after osmotic shift from isotonic (291 mOsm) to hypertonic (873 mOsm) solution.** A, B, C and D represent the changes of the normalized cell volume at 25, 15, 5 and −2°C, respectively.
(TIF)

Figure S2 **Volume changes of Sf21 cell after osmotic shift from CPA free isotonic solution to solution with 1.0 M glycerol.** A, B, C and D represent the changes of normalized cell volume at 25, 15, 5 and −2°C, respectively.
(TIF)

Figure S3 **Dependence of the glycerol permeability coefficient of Sf21 cell on the glycerol concentration at 25, 15, 5 and** −**2**°**C, respectively.**
(TIF)

Figure S4 **Predicted cell volume changes of Sf21 after various osmotic shifts at 25**°**C using the permeability coefficients measure by this study (from PBS to 1.0, 1.5 and 2.0 M glycerol solutions).** The dot and dash-dot lines represent the predictions with the hypothesized transport properties being independent of the glycerol concentration.
(TIF)
