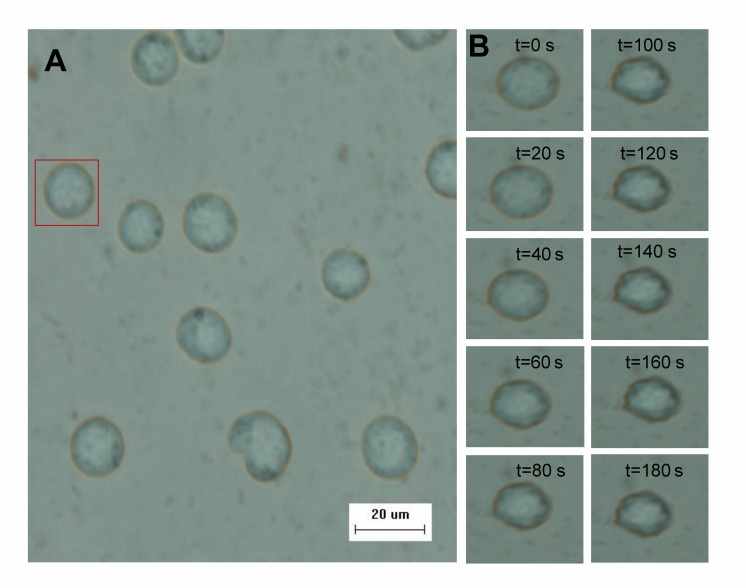# Correction: Dual Dependence of Cryobiogical Properties of Sf21 Cell Membrane on the Temperature and the Concentration of the Cryoprotectant

**DOI:** 10.1371/annotation/e12fcb03-9a8f-4fee-b95b-804d44e2285c

**Published:** 2013-10-30

**Authors:** Jianye Wang, Kaixuan Zhu, Gang Zhao, Jian Ren, Cui Yue, Dayong Gao

The images for Figures 1 and 2 were incorrectly reversed in the article. The legends are correct. The correct versions of these figures are available below.

Figure 1: 

**Figure:**
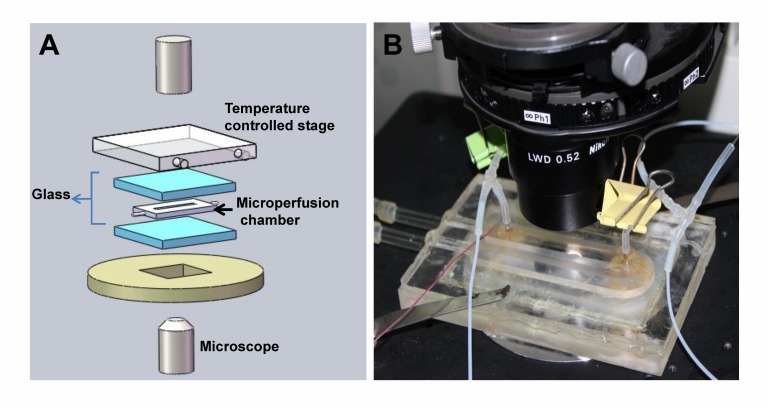


Figure 2: 

**Figure:**