# Supplementary figures and images for: Dual Dependence of Cryobiogical Properties of Sf21 Cell Membrane on the Temperature and the Concentration of the Cryoprotectant
Source: PLoS One. 2013 Sep 4;8(9):e72836. doi: 10.1371/journal.pone.0072836 (PMC3762842; doi:10.1371/journal.pone.0072836)

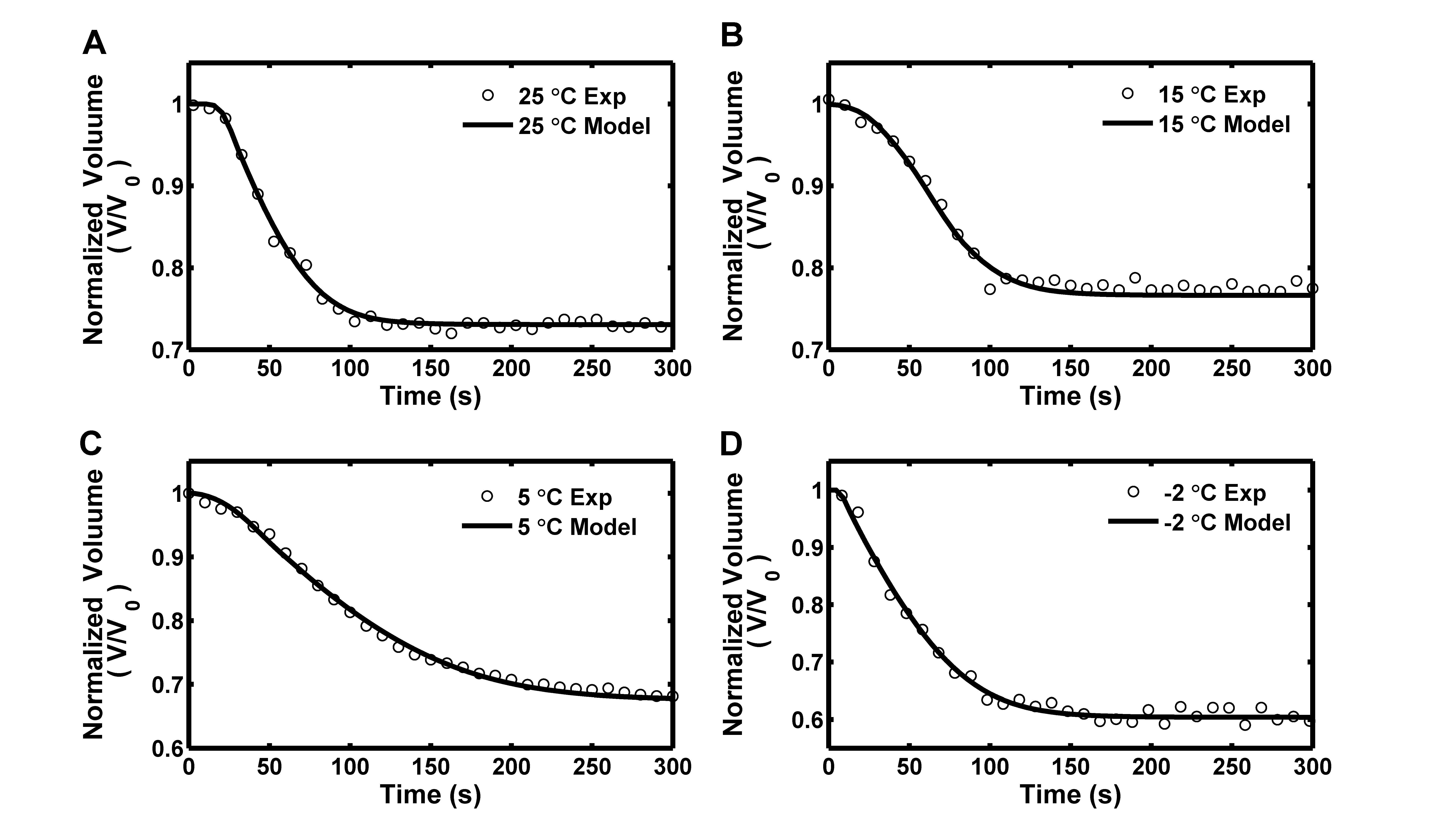

Supplement: Figure S1 — Volume responses of Sf21 cell after osmotic shift from isotonic (291 mOsm) to hypertonic (873 mOsm) solution. A, B, C and D represent the changes of the normalized cell volume at 25, 15, 5 and −2°C, respectively. (TIF) [file pone.0072836.s001.tif]

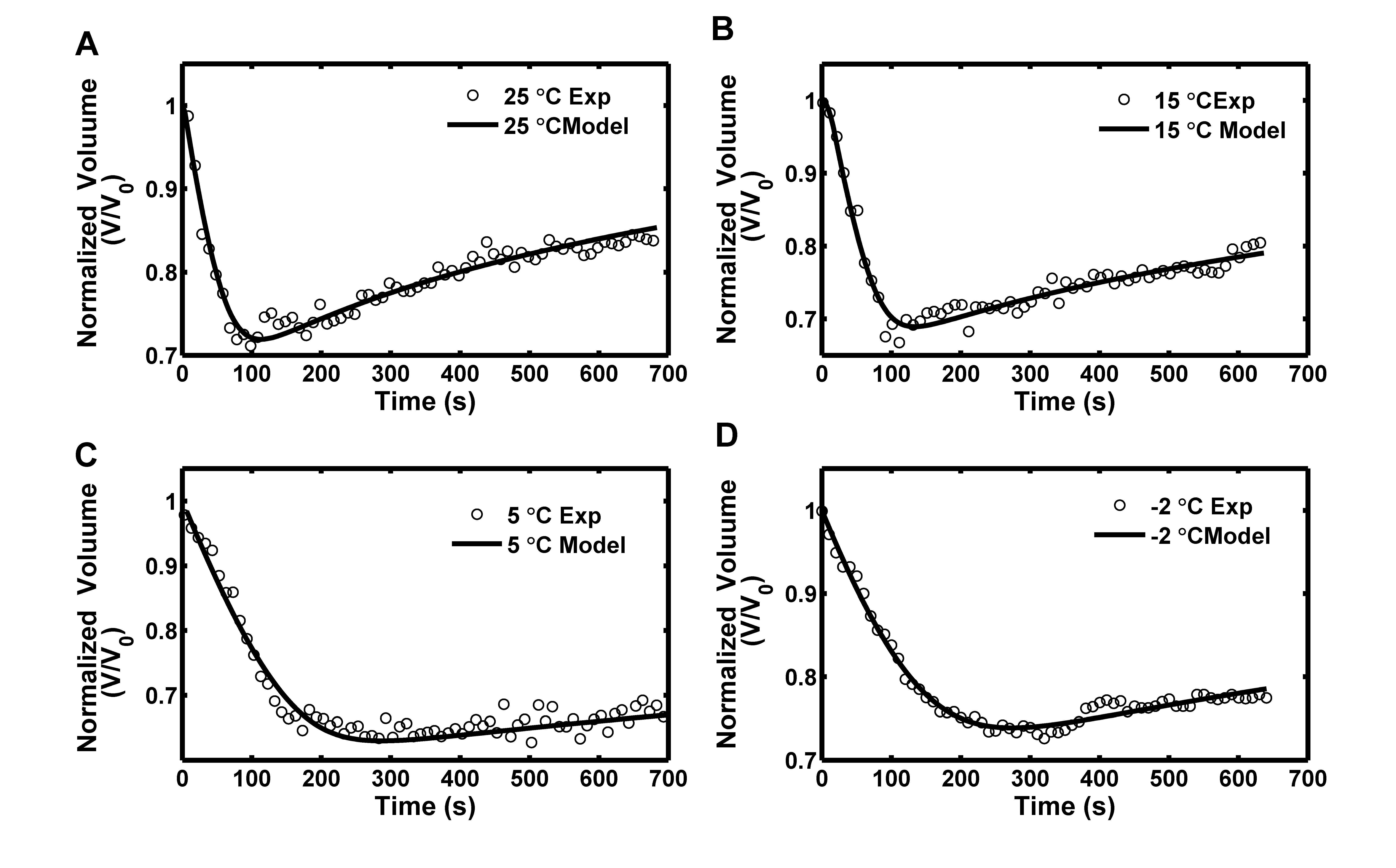

Supplement: Figure S2 — Volume changes of Sf21 cell after osmotic shift from CPA free isotonic solution to solution with 1.0 M glycerol. A, B, C and D represent the changes of normalized cell volume at 25, 15, 5 and −2°C, respectively. (TIF) [file pone.0072836.s002.tif]

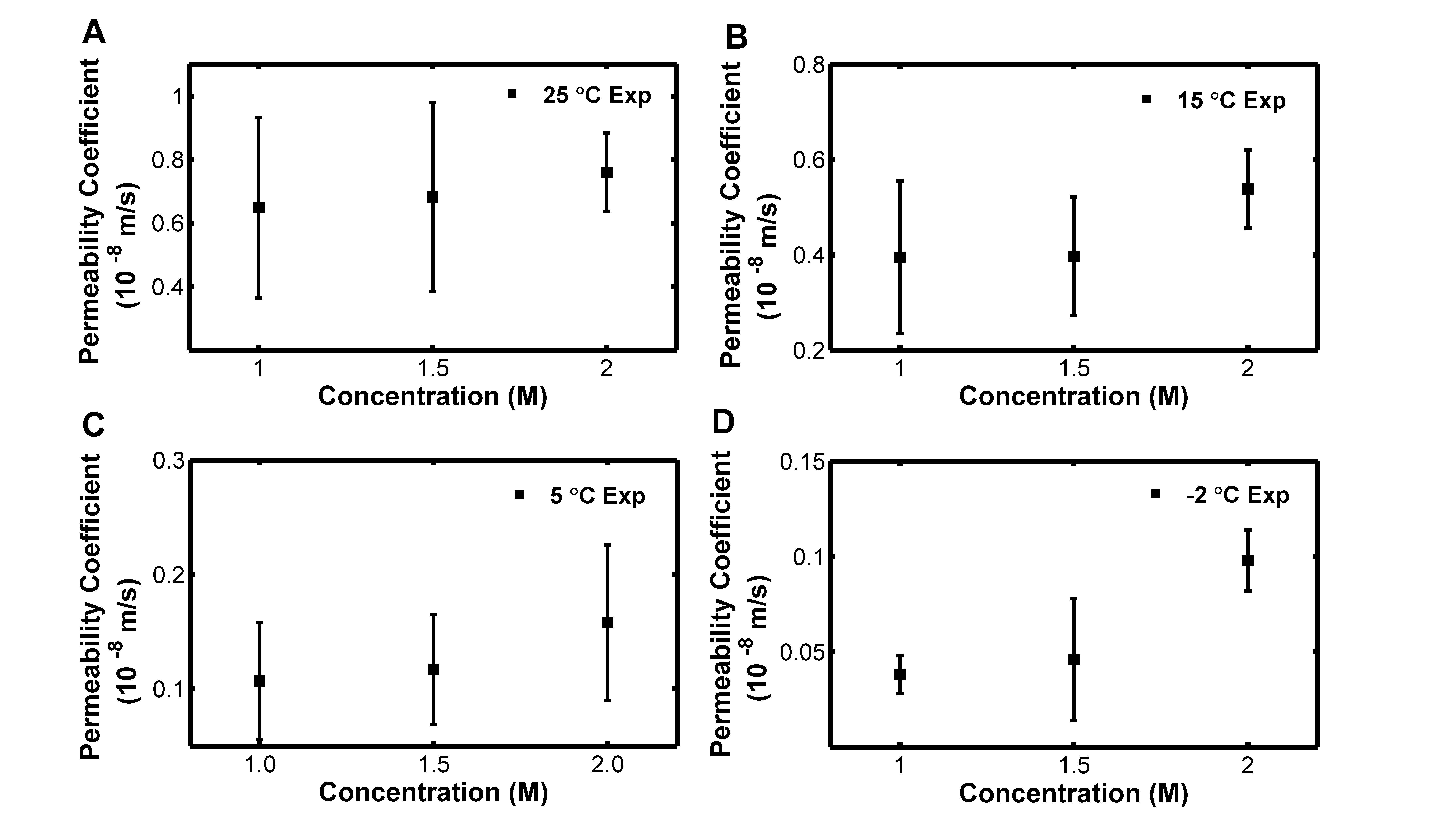

Supplement: Figure S3 — Dependence of the glycerol permeability coefficient of Sf21 cell on the glycerol concentration at 25, 15, 5 and −2°C, respectively. (TIF) [file pone.0072836.s003.tif]

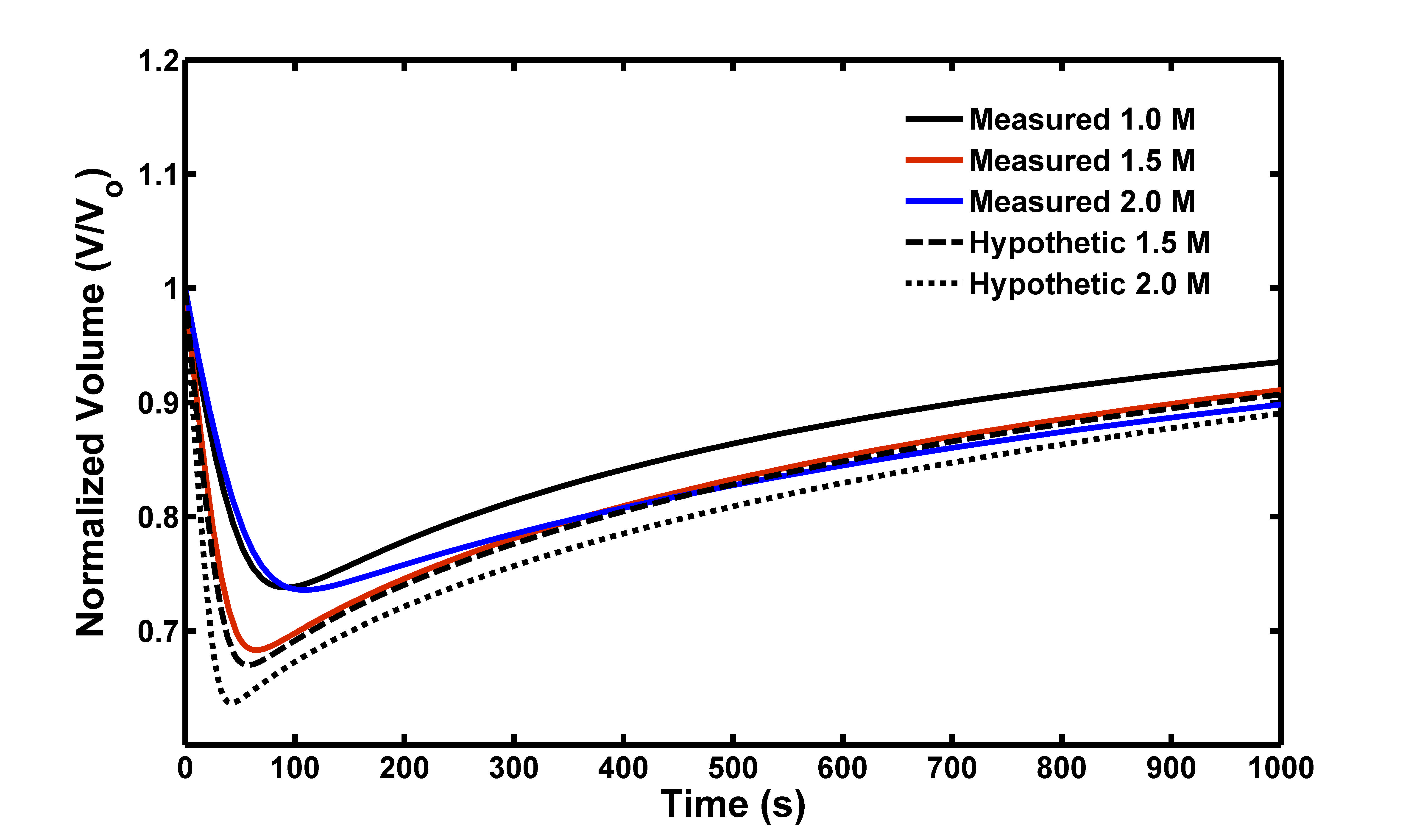

Supplement: Figure S4 — Predicted cell volume changes of Sf21 after various osmotic shifts at 25°C using the permeability coefficients measure by this study (from PBS to 1.0, 1.5 and 2.0 M glycerol solutions). The dot and dash-dot lines represent the predictions with the hypothesized transport properties being independent of the glycerol concentration. (TIF) [file pone.0072836.s004.tif]
